# Adverse Event Reporting in Randomized Clinical Trials for Multiple Myeloma

**DOI:** 10.1001/jamanetworkopen.2023.42195

**Published:** 2023-11-10

**Authors:** Mimi Najjar, John McCarron, Edward R. Scheffer Cliff, Katherine Berger, David P. Steensma, Samer Al Hadidi, Rajshekhar Chakraborty, Aaron Goodman, Eric Anto, Tom Greene, Douglas Sborov, Ghulam Rehman Mohyuddin

**Affiliations:** 1 Department of Oncology, Johns Hopkins School of Medicine, Baltimore, Maryland; 2 Division of Hematology and Hematological Malignancies, Huntsman Cancer Institute, University of Utah, Salt Lake City; 3 Program on Regulation, Therapeutics, and Law, Division of Pharmacoepidemiology and Pharmacoeconomics, Brigham and Women’s Hospital, Harvard Medical School, Boston, Massachusetts; 4 Independent Patient Advocate, Pawcatuck, Connecticut; 5 David P. Steensma LLC, Wellesley, Massachusetts; 6 Myeloma Center, Winthrop P. Rockefeller Cancer Institute, University of Arkansas for Medical Sciences, Little Rock; 7 Herbert Irving Comprehensive Cancer Center, Columbia University Irving Medical Center, New York, New York; 8 Division of Hematology, University of California, San Diego; 9 Division of Biostatistics, Department of Population Health Sciences, University of Utah, Salt Lake City

## Abstract

**Importance:**

Cancer treatment can result in burdensome toxic effects that profoundly affect patient quality of life. In seeking to emphasize the efficacy of tested treatments, clinical trial reports may use subjective or minimizing terms to describe adverse events (AEs).

**Objective:**

To evaluate patterns of AE reporting in multiple myeloma (MM) randomized clinical trials (RCTs) published between 2015 and early 2023.

**Design, Setting, and Participants:**

For this cohort study, the PubMed, Embase, and Cochrane Central Register of Controlled Trials databases were searched to assess the prevalence of minimizing terms in MM RCTs published between January 1, 2015, and March 1, 2023. Minimizing terms were defined as subjective terms used to favorably describe the safety profile of the intervention. The terms searched included *convenient*, *manageable*, *acceptable*, *expected*, *well-tolerated*, *tolerable*, *favorable*, and *safe*. Final data analysis was performed on July 21, 2023.

**Main Outcomes and Measures:**

The primary outcome was the occurrence of at least 1 minimizing term in an article. Univariate logistic regression analyses were performed to evaluate the association between the presence of at least 1 minimizing term and the actual incidence of grade 3 or 4 AEs, serious AEs, or grade 5 AEs.

**Results:**

Of the 65 RCTs included, 56 (86%) used minimizing terms when describing treatment-emergent AEs. The most frequently used minimizing terms were *well-tolerated* or *tolerable* in 29 trials (45%), *manageable* in 18 (28%), and *acceptable* in 16 (25%). Grade 3 or 4 AE rate in the examined RCTs ranged from 23% to 94%, with a median of 75% (IQR, 59%-82%). A univariate regression analysis demonstrated no association between the use of minimizing terms and grade 3 or 4 AE rates (odds ratio [OR], 1.35 [95% CI, 0.88-2.10] per 10% AE rate increase; *P* = .17) or grade 5 AE rates (OR, 3.16 [95% CI, 0.27-12.7] per 10% AE rate increase; *P* = .45).

**Conclusions and Relevance:**

These findings suggest that trial investigators and sponsors regularly use minimizing terms to describe toxic effects in MM trials, and use of this terminology may not reflect actual AE rates in these studies. Instead of using these terms, trial investigators should highlight event rates and patient-reported outcomes, to allow clinicians and patients to better evaluate the true tolerability of AEs.

## Key Points

**Question:**

Do reports of multiple myeloma (MM) randomized clinical trials (RCTs) downplay toxic effects with their choice of words?

**Findings:**

In this cohort study of 65 MM RCTs, 86% used minimizing terms when describing adverse events (AEs). There was no association between use of minimizing terms and grade 3 or 4 AE rate or toxic effect–related deaths (grade 5 AEs).

**Meaning:**

These findings suggest that trial investigators and sponsors regularly use minimizing terms to describe toxic effects in MM trials, and use of this terminology may not reflect actual AE rates.

## Teaser

This cohort study examines the use of minimizing terms in adverse event reporting in multiple myeloma randomized clinical trials published between 2015 and early 2023.

## Introduction

Dramatic advances in the treatment of multiple myeloma (MM) have led to improved outcomes.^1^ Nevertheless, disease relapse is common, and patients typically require long-term ongoing treatment. These treatments can result in considerable toxic effects that negatively affect patient quality of life.^2^ Multiple myeloma therapies, although effective, may be associated with unpleasant adverse effects ranging from weakness, fatigue, nausea, and diarrhea to more serious adverse events (SAEs) such as permanent neuropathy, cytopenias, life-threatening infections and bleeding events, and even death in some cases.^3,4,5,6,7,8^ In a disease such as MM in which treatment is often given continuously over longer periods (often for years, as maintenance therapy), toxic effects and tolerability become even more important.

Clinical trial efficacy and safety results are typically reported at scientific meetings and in peer-reviewed articles. Because efficacy is often preliminarily assessed in single-group phase 2 trials, how these trials are reported becomes increasingly critical. Furthermore, as more active therapies in MM become available, the ability to understand and compare their safety profiles is becoming increasingly important to inform patient decision-making. Clinical trial investigators may increasingly use phrases that seek to minimize toxic effects, which we refer to as *minimizing terms*, when reporting clinical trial safety results.^9^ Terms such as *tolerable*, *manageable*, and *safe* may be used in a subjective fashion that may not reflect the actual toxic effects of these interventions. In the absence of patient-reported outcomes (PROs), minimizing terms also may not reflect patient perceptions of whether these side effects are indeed tolerable or manageable. Patient-reported outcomes are ultimately the best way to know whether a given therapy’s toxic effects are acceptable or tolerable to patients.^10^

To our knowledge, no analysis to date has assessed the use of minimizing terms within a cohort of published randomized clinical trials (RCTs). In this study of MM RCTs published between 2015 and early 2023, we estimated the prevalence of the use of minimizing terms and explored the characteristics of studies that used these terms.

## Methods

For this cohort study, no direct patient information was obtained and the data used were gathered from publicly available and deidentified sources; therefore, this study was not submitted for institutional review board review in accordance with the Common Rule. The study followed the Strengthening the Reporting of Observational Studies in Epidemiology (STROBE) reporting guideline for observational studies.

### Data Sources

A clinical trials database and search strategy generated for a previous systematic review was used for this study.^11^ We performed a search of 3 databases: PubMed, Embase, and the Cochrane Central Register of Controlled Trials. A search strategy example using Embase is highlighted in the eMethods in Supplement 1. Briefly, the search strategy was designed to identify all MM RCTs published between January 1, 2015, and March 1, 2023. Although this search strategy included abstracts and conference proceedings, they were not included in our analysis because we limited final inclusion in this study to published articles. The search was last updated in March 1, 2023. The search strategy had no language restrictions. All other publication types, including editorials, case reports, case series, review articles, and case-control, retrospective or prospective cohort, or single-group studies, were excluded. Trials that had multiple adaptive randomizations with separate publications for each aspect of the trial were treated as separate trials.

### Study Selection

In our analysis, we included only initial study reports and excluded follow-up publications or post hoc analyses. Three investigators (M.N., J.M., and G.R.M.) conducted a thorough review of the original articles and were not blinded to the study hypothesis. The search terms were predetermined, and any disputes were reviewed by all 3 investigators for clarification. This study was not separately registered on PROSPERO, as it used a data set from a previous systematic review.^11^

We identified the following characteristics of the included studies: disease phase (upfront vs relapsed or refractory), study location (enrollment in 1 country vs multiple countries), and study sponsor (pharmaceutical funded vs cooperative or single-center study). We collected data on SAEs and grade 3 to 5 adverse events (AEs) from each study report as defined per each respective study. Whether PROs were presented in the publication was also recorded.

Minimizing terms were defined as subjective terms used to describe the safety profile of the intervention. We specifically looked for the terms *manageable*, *acceptable*, *expected*, *well-tolerated*, *tolerable*, *favorable*, *convenient*, and *safe*. Alternate spellings (eg, *favourable*) were also included.

### Statistical Analysis

The primary study outcome was the occurrence of at least 1 minimizing term in an article. We performed univariate logistic regression analyses to evaluate the association between the presence of at least 1 minimizing term and the actual incidence of grade 3 or 4 AEs, SAEs, or grade 5 AEs. Each logistic regression was restricted to articles in which the exposure variable (incidence of grade 3 or 4 AEs, SAEs, and grade 5 AEs) was nonmissing. Using the Fisher exact test of independence, we explored the association between use of at least 1 minimizing term and industry funding as well as whether PROs were reported in the article.

Statistical analysis was carried out using R, version 4.2.2 (R Project for Statistical Computing) via the RStudio integrated development environment. In addition to the core functionality of R, we used the gtsummary package, which enhances table generation and summary statistics presentation. We used 2-sided hypothesis tests, with a significance level of 5% established a priori. This significance level was used to assess the results of hypothesis tests, confidence, and any other statistical inferences made throughout the analysis. Final data analysis was performed on July 21, 2023.

## Results

**Figure 1.**
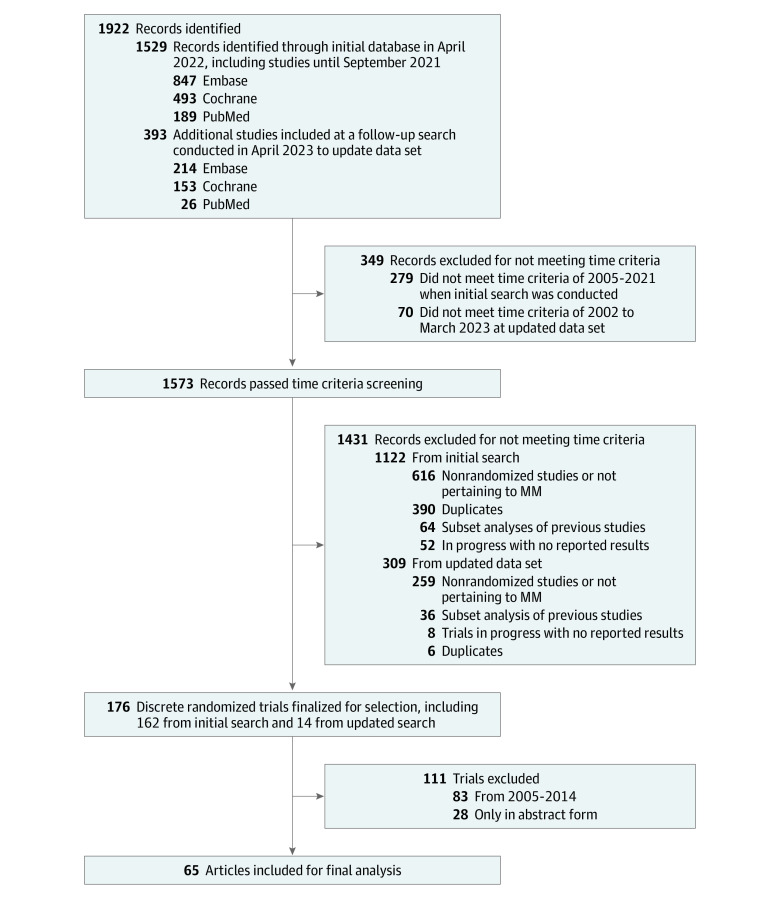
Flow Diagram Depicting the Search Strategy and Study Inclusion MM indicates multiple myeloma.

After excluding duplicate reports for the same trial and trials that did not meet the inclusion criteria, 65 discrete RCTs^12,13,14,15,16,17,18,19,20,21,22,23,24,25,26,27,28,29,30,31,32,33,34,35,36,37,38,39,40,41,42,43,44,45,46,47,48,49,50,51,52,53,54,55,56,57,58,59,60,61,62,63,64,65,66,67,68,69,70,71,72,73,74,75,76^ were identified (Figure 1). The Table highlights characteristics of the included studies.

**Table.** Characteristics of Included Studies

Study characteristic	No. (%) of studies (N = 65)
Sponsor	
Industry	37 (57)
Nonindustry	28 (43)
Enrollment location	
Multiple countries	39 (60)
Limited to the US	10 (15)
Limited to a single country other than the US	16 (25)
Maintenance	5 (8)
2-Drug vs 3-drug	22 (34)

### Frequency of Minimizing Terms

**Figure 2.**
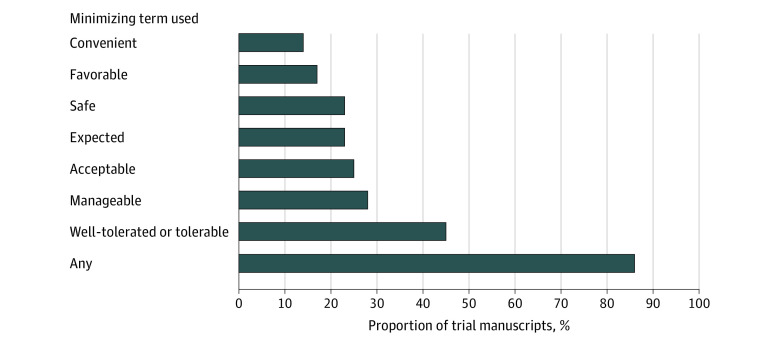
Published Articles on Multiple Myeloma Randomized Clinical Trials Using Minimizing Terms

Of the 65 trials in this study, 56 (86%) used minimizing terms. The most frequently used minimizing terms were *well-tolerated* or *tolerable*, as observed in 29 trials (45%). Additionally, 18 trials (28%) used *manageable*, 16 (25%) used *acceptable*, 15 (23%) used *expected*, 15 (23%) used *safe*, 11 (17%) used *favorable*, and 9 (14%) used *convenient* (Figure 2).

We performed an additional analysis to determine whether minimizing terms were used to describe a specific toxic effect or the general safety profile of a treatment approach. Of the 56 trials that used minimizing terms, 44 (78%) used at least 1 minimizing term that referred to toxic effects in a general sense, 11 (20%) used them to describe both the toxic effects of treatment generally and of a specific toxic effect, and 1 (2%) used them only to describe a specific toxic effect. The eTable in Supplement 1 highlights individual studies and the respective toxic effects and terms used.

### AE Reporting

The frequency of SAEs was reported in 38 trials (59%). Serious adverse event rates ranged from 19% to 66% (median, 48% [IQR, 35%-56%]). There were 33 trials (87%) that used at least 1 minimizing term (median SAE rate, 48% [IQR, 34%-55%]) and 5 trials (13%) that did not use any (median SAE rate, 47% [IQR, 45%-63%]).

The overall frequency of grade 3 or 4 AEs was reported in 37 trials (57%). Grade 3 or 4 AE rates in these trials ranged from 23% to 94% (median, 75% [IQR, 59%-82%]). There were 31 trials (84%) that used at least 1 minimizing term (median grade 3 or 4 AE rate, 75% [IQR, 63%-82%]) and 6 (16%) that did not use any (median grade 3 or 4 AE rate, 57% [IQR, 36%-78%]).

The frequency of grade 5 (fatal) AEs was reported in 45 trials (69%). Grade 5 AE rates ranged from 0% to 19% (median, 3% [IQR, 1%-7%]). There were 40 trials (89%) that used at least 1 minimizing term (median grade 5 AE rate, 3% [IQR, 1%-7%]) and 5 (11%) that did not use any (median grade 5 AE rate, 1% [IQR, 1%-4%]). Among the 40 studies in which minimizing terms were used and grade 5 AEs occurred, these terms were used to describe the general treatment profile in 30 trials (75%) and were used for both general treatment safety and a specific toxic effect in 10 trials (25%). None of the minimizing terms used to describe a specific toxic effect referred to grade 5 AEs.

### Association Between AE Rates and Use of Minimizing Terms

In univariate regression analysis, no association was observed between grade 3 or 4 AE rate and whether a study used minimizing terms (odds ratio [OR], 1.35 [95% CI, 0.88-2.10] per 10% AE rate increase; *P* = .17). Furthermore, no association was observed between use of minimizing terms and grade 5 AE rate (OR, 3.16 [95% CI, 0.27-12.7] per 10% AE rate increase; *P* = .45) or SAE rate (OR, 0.68 [95% CI, 0.26-1.42] per 10% SAE rate decrease; *P* = .35).

### Association Between Industry Funding or PRO Reporting and Use of Minimizing Terms

This study included 37 industry-sponsored trials and 28 non–industry-sponsored trials. Using the Fisher exact test of independence, we explored the association between the use of at least 1 minimizing term and whether the study was industry sponsored. Among the 37 industry-sponsored studies, a higher proportion (35 [95%]) used minimizing terms. Comparatively, 21 of 28 non–industry-sponsored studies (75%) also used minimizing terms. An association was observed between the use of at least 1 minimizing term and whether a study was industry sponsored (OR, 5.68 [95% CI, 1.05-41.0]; *P* = .03).

We also used the Fisher exact test of independence to explore the association between the use of at least 1 minimizing term and whether the study reported PROs. Although a higher proportion of studies that did not report PROs used minimizing terms (37 of 40 [93%]) compared with those that reported PROs (19 of 25 [76%]), this difference was not statistically significant (OR, 0.26 [95% CI, 0.05-1.34]; *P* = .08).

## Discussion

In this study of MM RCTs, we observed that most studies (86%) used minimizing terms that did not necessarily reflect the actual toxic effects of the interventions being studied. Furthermore, the use of these terms was not associated with the actual frequency of SAEs, grade 3 or 4 AEs, or grade 5 (fatal) AEs related to the treatment being studied. The industry studies examined were more likely to use minimizing terms. Only a minority of studies used PROs, yet the use of minimizing terms was widespread. Our work highlights the inherent subjectivity and lack of clarity in how toxic effects are defined and reported in the current literature.

This study builds on and updates previous work that analyzed RCTs in 5 high-impact oncology journals across all cancer types in 2018.^9^ The investigators analyzed 122 trials and observed that 53 (43%) contained minimizing terms, substantially lower than the 86% observed in this study. By systematically analyzing all RCTs for a specific disease across all journals, we captured publications that may not have been subject to as rigorous or consistent a review or editing process as those in top journals. In this study, we also analyzed a longer, more recent period of trials (2015 to early 2023), highlighting that this problem continues despite awareness being raised several years ago. Furthermore, we characterized the attributes of studies that use minimizing terms and, to our knowledge, are the first to highlight that these terms were not associated with actual toxic effect rates or even treatment-related mortality. We also explored the association between use of minimizing terms and industry funding and PRO usage, both of which have important implications for future work in this space.

Our findings are especially important given that use of minimizing terms was widespread, despite scant usage of PROs.^10^ Compared with investigator assessments, PROs allow patients to decide whether they consider a treatment safe or tolerable. Given that AEs tend to be underreported by clinicians compared with patients,^77^ we must ask our patients if we really want to know the true toxicity profiles of our treatments. A larger sample size may reinforce our findings regarding the association between PROs and use of minimizing terms. Future trials can help address this problem by consistently reporting PROs, describing toxic effects objectively as they occur, and avoiding overemphasis of AE attribution and use of minimizing terms. Education for medical writers, who are often employed to draft reports of industry-sponsored trials, may help to curb overuse of minimizing terms.^78^

Examples of studies in which toxic effects were described without minimizing terms include trials of daratumumab and elotuzumab. For example, investigators in 1 study transparently reported that daratumumab use was associated with infusion-related reactions and a higher rate of neutropenia than the control therapy, instead of using minimizing terms.^79^ Another example of a trial in which toxic effects were transparently reported involved elotuzumab use in relapsed MM.^80^ Such language is commendable and an example for other trials to emulate.

### Limitations

This study has some limitations. Because we only analyzed RCTs, this phenomenon may be more common in early-phase nonrandomized trials and in conference presentations, which deserves further evaluation. Many studies did not report SAE rates or total grade 3 or 4 AE rates; this is a noteworthy finding that limited the statistical power of our analysis and may have led to selection bias. Given the high prevalence of minimizing terms, with the majority of articles using at least 1, our analysis had low statistical power to detect associations with various exposure variables. Thus, there may have been an association between rates of total or grade 3 or 4 AEs and use of minimizing terms that was not detected. There was no evaluation of use of other terms to describe toxic effects, and articles that used minimizing terms may also concurrently use other terms to describe such effects. The attribution of toxic effects (and death) to an intervention is inherently subjective and imperfect.^5,81^ Our review may not have included all separate publications that contained PROs. In some cases, the term *convenient* may be reasonable, such as when describing the administration routes.

## Conclusions

In this cohort study, we observed that trial investigators and sponsors regularly used minimizing terms to describe toxic effects in MM trials, especially in industry-sponsored studies, and this descriptive terminology did not reflect the actual rates of severe AEs or deaths in these trials. Instead of using these terms, trial investigators should highlight event rates and PROs to allow clinicians and patients to better evaluate the true tolerability of AEs.

## Notes

Supplement 1. **eTable.** Included Randomized Clinical Trials **eMethods.** Example Search Strategy on Embase

Supplement 2.
Data Sharing Statement
