# Supplementary material for: Adverse Event Reporting in Randomized Clinical Trials for Multiple Myeloma
Source: JAMA Netw Open. 2023 Nov 10;6(11):e2342195. doi: 10.1001/jamanetworkopen.2023.42195 (PMC10638643; doi:10.1001/jamanetworkopen.2023.42195)
Supplement: Supplement 1. — eTable. Included Randomized Clinical Trials eMethods. Example Search Strategy on Embase [file jamanetwopen-e2342195-s001.pdf]

Supplementary Online Content

Najjar M, McCarron J, Cliff ERS, et al. Adverse event reporting in randomized clinical trials for multiple myeloma. *JAMA Netw Open*. 2023;6(11):e2342195. doi:10.1001/jamanetworkopen.2023.42195

eTable. Included Randomized Clinical Trials

eMethods. Example Search Strategy on Embase

This supplementary material has been provided by the authors to give readers additional information about their work.

© 2023 Najjar M et al. *JAMA Network Open*.

eTable. Included Randomized Clinical Trials

Trial name	Trial first author	Trial NCT	Uses minimizing terms (Yes/No?)	Minimizing term(s) used	Do the minimizing terms refer to the overall treatment approach, a specific toxicity or both?	Which minimizing term was used to describe a specific toxicity?	What was the specific toxicity?
Efficacy and safety of three bortezomib-based combinations in elderly, newly diagnosed multiple myeloma patients: Results from all randomized patients in the community-based, phase 3b UPFRONT study	Ruben Niesvizky	NCT00507416	No	NA	NA	NA	NA
Elotuzumab in combination with lenalidomide and dexamethasone in patients with relapsed multiple myeloma: final phase 2 results from the randomised, open-label, phase 1b-2 dose-escalation study.	Paul G Richardson	NCT00742560	Yes	Well tolerated, Acceptable	General	NA	NA
Bortezomib, cyclophosphamide, dexamethasone versus lenalidomide, cyclophosphamide, dexamethasone in multiple myeloma patients at first relapse	Vittorio Montefusco	NA	Yes	Acceptable	General	NA	NA
Daratumumab plus Bortezomib, Melphalan, and Prednisone for Untreated Myeloma	M.-V. Mateos	NCT02195479	No	NA	NA	NA	NA
Pembrolizumab plus lenalidomide and dexamethasone for patients with treatment-naive multiple myeloma (KEYNOTE-185): a randomised, open-label, phase 3 trial	Saad Zafar Usmani	NCT02579863	Yes	Acceptable	General	NA	NA
Autologous Transplantation, Consolidation, and Maintenance Therapy in Multiple Myeloma: Results of the BMT CTN 0702 Trial.	Edward A. Stadtmauer	NCT01109004	No	NA	NA	NA	NA
Randomized phase III study (ADMYRE) of plitidepsin in combination with dexamethasone vs. dexamethasone alone in patients with relapsed/refractory multiple myeloma	Ivan Spicka	NCT01102426	Yes	Acceptable	General	NA	NA
Bortezomib, lenalidomide, and dexamethasone as induction therapy prior to autologous transplant in multiple myeloma	Laura Rosiñol	NCT01916252	Yes	Well tolerated	General	NA	NA
Bortezomib, thalidomide, and dexamethasone with or without daratumumab before and after autologous stem-cell transplantation for newly diagnosed multiple myeloma (CASSIOPEIA): a randomised, open-label, phase 3 study	Philippe Moreau	NCT02541383	Yes	Acceptable, Manageable, Well tolerated	General	NA	NA
Pomalidomide, bortezomib, and dexamethasone for patients with relapsed or refractory multiple myeloma previously treated with lenalidomide (OPTIMISMM): a randomised, open-label, phase 3 trial	Paul G Richardson	NCT01734928	Yes	Encouraging	General	NA	NA
Pembrolizumab plus pomalidomide and dexamethasone for patients with relapsed or refractory multiple myeloma (KEYNOTE-183): a randomised, open-label, phase 3 trial.	Maria-Victoria Mateos	NCT02576977	Yes	Expected, Manageable, Well tolerated	General	NA	NA

© 2023 Najjar M et al. *JAMA Network Open*.

Daratumumab, Lenalidomide, and Dexamethasone for Multiple Myeloma	Meletios A. Dimopoulos	NCT02076009	Yes	Well tolerated	General	NA	NA
Lenalidomide maintenance versus observation for patients with newly diagnosed multiple myeloma (Myeloma XI): a multicentre, open-label, randomised, phase 3 trial. ALSO Response-adapted intensification with cyclophosphamide, bortezomib, and dexamethasone versus no intensification in patients with newly diagnosed multiple myeloma (Myeloma XI): a multicentre, open-label, randomised, phase 3 trial	Graham H Jackson	NA	Yes	Well tolerated	General	NA	NA
Phase 3 study of subcutaneous bortezomib, thalidomide, and prednisolone consolidation after subcutaneous bortezomib-based induction and autologous stem cell transplantation in patients with previously untreated multiple myeloma: the VCAT study	Noemi Horvath	NCT01539083	Yes	Manageable	General	NA	NA
Carfilzomib or bortezomib with melphalan-prednisone for transplant-ineligible patients with newly diagnosed multiple myeloma	Thierry Facon	NCT01818752	Yes	Acceptable, well tolerated	General	NA	NA
Daratumumab plus Lenalidomide and Dexamethasone for Untreated Myeloma.	T. Facon	NCT02252172	No	NA	NA	NA	NA
Elotuzumab Therapy for Relapsed or Refractory Multiple Myeloma	Sagar Lonial	NCT01239797	Yes	No significant effect on quality of life	General	NA	NA
Carfilzomib and dexamethasone versus bortezomib and dexamethasone for patients with relapsed or refractory multiple myeloma (ENDEAVOR): a randomised, phase 3, open-label, multicentre study	Meletios A Dimopoulos	NCT01568866	Yes	Well tolerated, Aceptable, Manageable	Both	Manageable	Hypertension
Conditioning with busulfan plus melphalan versus melphalan alone before autologous haemopoietic cell transplantation for multiple myeloma: an open-label, randomised, phase 3 trial	Qaiser Bashir	NCT01413178	Yes	Expected, Safe	Both	Expected	Mucosistis
Retreatment and prolonged therapy with subcutaneous bortezomib in patients with relapsed multiple myeloma: A randomized, controlled, phase III study.	Evangelos Terpos	NCT01910987	Yes	Expected, tolerable, Safe	General	NA	NA
Daratumumab plus bortezomib and dexamethasone versus bortezomib and dexamethasone in relapsed or refractory multiple myeloma	Antonio Palumbo	NCT02136134	Yes	Expected, Acceptable	Both	Expected	Infusion Reaction
Once weekly versus twice weekly carfilzomib dosing in patients with relapsed and refractory multiple myeloma (A.R.R.O.W.): interim analysis results of a randomised, phase 3 study	Philippe Moreau	NCT02412878	Yes	Well tolerated, Favourable, Safe	General	NA	NA
Elotuzumab plus Pomalidomide and Dexamethasone for Multiple Myeloma	Meletios A. Dimopoulos	NCT02654132	Yes	Favourable	General	NA	NA

© 2023 Najjar M et al. *JAMA Network Open*.

A randomized phase III study of carfilzomib vs low-dose corticosteroids with optional cyclophosphamide in relapsed and refractory multiple myeloma (FOCUS)	R Hájek	NCT01302392	Yes	Expected	General	NA	NA
Response-adapted lenalidomide maintenance in newly diagnosed, transplant-eligible multiple myeloma: Results from the multicenter phase III GMMG-MM5 Trial	Hartmut Goldschmidt	NA	Yes	Manageable	General	NA	NA
Bortezomib with lenalidomide and dexamethasone versus lenalidomide and dexamethasone alone in patients with newly diagnosed myeloma without intent for immediate autologous stem-cell transplant (SWOG S0777): a randomised, open-label, phase 3 trial	Brian G M Durie	NCT00644228	Yes	Expected, Acceptable, Favourable	Both	Expected	Neurological toxicity
Lenalidomide, bortezomib, and dexamethasone with transplantation for myeloma	Michel Attal	NCT01191060	No	NA	NA	NA	NA
Melphalan, prednisone, and lenalidomide versus melphalan, prednisone, and thalidomide in untreated multiple myeloma.	Sonja Zweegman	NA	Yes	Manageable	Specific	Manageable	Hematologic safety profile
Oral Ixazomib, lenalidomide, and dexamethasone for multiple myeloma	P. Moreau	NCT01564537	Yes	Acceptable, Manageable	Both	Manageable	GI events
VTD is superior to VCD prior to intensive therapy in multiple myeloma: results of the prospective IFM2013-04 trial	Philippe Moreau	NCT01564537	Yes	Manageable	General	NA	NA
Triplet vs doublet lenalidomide-containing regimens for the treatment of elderly patients with newly diagnosed multiple myeloma	Valeria Magarotto	NCT01093196	Yes	Well tolerated	General	NA	NA
Randomized phase III trial of consolidation therapy with bortezomib-lenalidomide-Dexamethasone (VRd) vs bortezomib-dexamethasone (Vd) for patients with multiple myeloma who have completed a dexamethasone based induction regimen.	SJ Jacobus	NA	No	NA	NA	NA	NA
Phase 3 trial of three thalidomide-containing regimens in patients with newly diagnosed multiple myeloma not transplant-eligible	V. T. M. Hungria	(NCT01532856	Yes	Attractive, Safe	General	NA	NA
Phase III trial of bortezomib, cyclophosphamide and dexamethasone (VCD) versus bortezomib, doxorubicin and dexamethasone (PAd) in newly diagnosed myeloma	E K Mai	NA	Yes	Favourable, Safe	General	NA	NA
Chemotherapy plus lenalidomide versus autologous transplantation, followed by lenalidomide plus prednisone versus lenalidomide maintenance, in patients with multiple myeloma: A randomised, multicentre, phase 3 trial	Francesca Gay	NCT01091831	Yes	Well tolerated, Expected, Manageable	General	NA	NA
Daratumumab, lenalidomide, bortezomib, and dexamethasone for transplant-eligible newly diagnosed multiple myeloma: the GRIFFIN trial	Peter M Voorhees	NCT02874742	Yes	Well tolerated, Acceptable, Manageable, Safe	Both	Manageable	Infection risk
Subcutaneous versus intravenous daratumumab in patients with relapsed or refractory multiple myeloma (COLUMBA): a multicentre, open-label, non-inferiority, randomised, phase 3 trial	Maria-Victoria Mateos	NCT03277105	Yes	Well tolerated, acceptable,	Both	Well tolerated	Infusion related

© 2023 Najjar M et al. *JAMA Network Open*.

				favourable, Safe			reaction
Autologous haematopoietic stem-cell transplantation versus bortezomib–melphalan–prednisone, with or without bortezomib–lenalidomide–dexamethasone consolidation therapy, and lenalidomide maintenance for newly diagnosed multiple myeloma (EMN02/HO95): a multicentre, randomised, open-label, phase 3 study	Michele Cavo	NCT01208766	No	NA	NA	NA	NA
Venetoclax or placebo in combination with bortezomib and dexamethasone in patients with relapsed or refractory multiple myeloma (BELLINI): a randomised, double-blind, multicentre, phase 3 trial	Shaji K Kumar	NCT02755597	Yes	Acceptable, Tolerable, Safe	General	NA	NA
Elotuzumab plus lenalidomide/dexamethasone (ELD) vs LD in patients with newly diagnosed multiple myeloma: Phase 2, randomized, open-label study in Japan	Kohmei Kubo	NCT02272803	Yes	Acceptable, Well Tolerated	General	NA	NA
Ixazomib-Thalidomide-low dose dexamethasone induction followed by maintenance therapy with ixazomib or placebo in newly diagnosed multiple myeloma patients not eligible for autologous stem cell transplantation; results from the randomized phase II HOVON-126/NMSG 21.13 trial	Sonja Zweegman	NA	Yes	Well tolerated	General	NA	NA
Salvage Autologous Transplant and Lenalidomide Maintenance Versus Continuous Lenalidomide/Dexamethasone for Relapsed Multiple Myeloma: Results of the Randomized GMMG Phase III Multicenter Trial Relapse	Hartmut Goldschmidt	NA	Yes	Expected	General	NA	NA
Randomized phase 2 study: elotuzumab plus bortezomib/dexamethasone vs bortezomib/dexamethasone for relapsed/refractory MM	Andrzej Jakubowiak	NCT01478048	Yes	Well tolerated	General	NA	NA
Randomized multicenter phase 2 study of pomalidomide, cyclophosphamide, and dexamethasone in relapsed refractory myeloma	Rachid C Baz	NCT01432600	Yes	Well tolerated	General	NA	NA
A randomized study of melphalan 200 mg/m(2) vs 280 mg/m(2) as a preparative regimen for patients with multiple myeloma undergoing auto-SCT	W I Bensinger	NCT00217438	Yes	Well tolerated, Expected, Manageable	Both	Manageable	Infusion toxicities
Isatuximab plus pomalidomide and low-dose dexamethasone versus pomalidomide and low-dose dexamethasone in patients with relapsed and refractory multiple myeloma (ICARIA-MM): a randomised, multicentre, open-label, phase 3 study.	Michel Attal	NCT02990338	Yes	Well tolerated	General	NA	NA
Isatuximab, carfilzomib, and dexamethasone in relapsed multiple myeloma (IKEMA): a multicentre, open-label, randomised phase 3 trial	Philippe Moreau	NCT03275285	Yes	Expected, Manageable	General	NA	NA

© 2023 Najjar M et al. *JAMA Network Open*.

Carfilzomib, dexamethasone, and daratumumab versus carfilzomib and dexamethasone for patients with relapsed or refractory multiple myeloma (CANDOR): results from a randomised, multicentre, open-label, phase 3 study	Meletios Dimopoulos	NCT03158688	Yes	Favourable	General	NA	NA
Ixazomib as Postinduction Maintenance for Patients With Newly Diagnosed Multiple Myeloma Not Undergoing Autologous Stem Cell Transplantation: the Phase III TOURMALINE-MM4 Trial	Meletios A Dimopoulos	NCT02312258	Yes	Well tolerated, Expected, Favourable	General	NA	NA
Daratumumab plus pomalidomide and dexamethasone versus pomalidomide and dexamethasone alone in previously treated multiple myeloma (APOLLO): an open-label, randomised, phase 3 trial	Meletios A Dimopoulos	NCT03180736	Yes	Manageable, Convenient	Both	Manageable	Neutropenia and infections
Once-per-week selinexor, bortezomib, and dexamethasone versus twice-per-week bortezomib and dexamethasone in patients with multiple myeloma (BOSTON): a randomised, open-label, phase 3 trial	Sebastian Grosicki	NCT03110562	Yes	Manageable, Convenient	General	NA	NA
Carfilzomib or bortezomib in combination with lenalidomide and dexamethasone for patients with newly diagnosed multiple myeloma without intention for immediate autologous stem-cell transplantation (ENDURANCE): a multicentre, open-label, phase 3, randomised, controlled trial	Shaji K Kumar	NCT01863550	No	NA	NA	NA	NA
Efficacy and Tolerability of High- versus Low-dose Lenalidomide Maintenance Therapy of Multiple Myeloma after Autologous Blood Stem Cell Transplantation	Roland Fenk	NA	Yes	Manageable	General	NA	NA
Daratumumab, Bortezomib, and Dexamethasone Versus Bortezomib and Dexamethasone in Chinese Patients with Relapsed or Refractory Multiple Myeloma: Phase 3 LEPUS (MMY3009) Study	Jin Lu	NCT03234972	Yes	Consistent	General	NA	NA
Efficacy and safety of oral panobinostat plus subcutaneous bortezomib and oral dexamethasone in patients with relapsed or relapsed and refractory multiple myeloma (PANORAMA 3): an open-label, randomised, phase 2 study	Jacob P Laubach	NCT02654990	Yes	Expected, Favourable	General	NA	NA
Bortezomib, Melphalan, and Prednisone With or Without Daratumumab in Transplant-ineligible Asian Patients With Newly Diagnosed Multiple Myeloma: The Phase 3 OCTANS Study	Dominik Dytfeld	NCT02659293	Yes	Manageable, Consistent	General	NA	NA
Carfilzomib, lenalidomide, dexamethasone, and cyclophosphamide (KRdc) as induction therapy for transplant-eligible, newly diagnosed multiple myeloma patients (Myeloma XI+): Interim analysis of an open-label randomised controlled trial	Graham H Jackson	NA	Yes	Well tolerated, Safe	General	NA	NA
Dose/schedule-adjusted Rd-R vs continuous Rd for elderly, intermediate-fit patients with newly diagnosed multiple myeloma	Alessandra Larocca	NCT02215980	Yes	Well tolerated, Safe	General	NA	NA
Addition of isatuximab to lenalidomide, bortezomib, and dexamethasone as induction therapy for newly diagnosed, transplantation-eligible patients with multiple myeloma (GMGG-HD7): part 1 of	Hartmut Goldschmidt	NCT03617731.	Yes	Expected, Safe	Both	Expected	Neutropenia

© 2023 Najjar M et al. *JAMA Network Open*.

an open-label, multicentre, randomised, active-controlled, phase 3 trial							
The addition of vorinostat to lenalidomide maintenance for patients with newly diagnosed multiple myeloma of all ages: results from 'Myeloma XI', a multicentre, open-label, randomised, phase III trial	Dominik Dytfeld	NCT02659293	Yes	Well tolerated, Manageable	General	NA	NA
Triplet Therapy, Transplantation, and Maintenance until Progression in Myeloma	Paul G Richardson	NCT01208662	No	NA	NA	NA	NA
Bortezomib and high-dose melphalan conditioning regimen in frontline multiple myeloma: an IFM randomized phase 3 study	Murielle Roussel	NCT02197221	Yes	Well tolerated, Expected, Safe	Both	Expected	Neurological toxicity
Melflufen or pomalidomide plus dexamethasone for patients with multiple myeloma refractory to lenalidomide (OCEAN): a randomised, head-to-head, open-label, phase 3 study	Fredrik H Schjesvold	NCT03151811	Yes	Well tolerated, Manageable, Safe	General	NA	NA
Ixazomib, lenalidomide and dexamethasone consolidation with randomized ixazomib or lenalidomide maintenance after autologous transplant in newly diagnosed multiple myeloma	Michael Slade	NCT02253316	Yes	Well tolerated, Favourable, Safe	General	NA	NA
Carfilzomib, lenalidomide, and dexamethasone or lenalidomide alone as maintenance therapy after autologous stem-cell transplantation in patients with multiple myeloma (ATLAS): interim analysis of a randomised, open-label, phase 3 trial	Dominik Dytfeld	NCT02659293	Yes	Well tolerated, Acceptable, Expected, Safe	General	NA	NA

© 2023 Najjar M et al. *JAMA Network Open*.

eMethods. Example Search Strategy on Embase:

Terms for supplement:

Myeloma OR myelomas OR Myelomatos* OR Kahler-Disease

"Multiple Myeloma"[Mesh]
"Clinical Trial, Phase III" [Publication Type]
"Clinical Trials, Phase III as Topic"[Mesh]
(Randomized controlled trial[pt] OR controlled clinical trial[pt] OR randomized[tiab] OR placebo[tiab] OR drug therapy[sh] OR randomly[tiab] OR trial[tiab] OR groups[tiab] NOT (animals [mh] NOT humans [mh]))

'myeloma'/exp
'phase 3 clinical trial'/exp
'phase 3 clinical trial (topic)'/exp
'Crossover procedure':de OR 'double-blind procedure':de OR 'randomized controlled trial':de OR ‘single-blind procedure':de OR (random* OR factorial* OR crossover* OR cross NEXT/1 over* OR placebo* OR doubl* NEAR/1 blind* OR singl* NEAR/1 blind* OR assign* OR allocat* OR volunteer*):de,ab,ti

© 2023 Najjar M et al. *JAMA Network Open*.
