# Supplementary material for: Adverse Event Reporting in Randomized Clinical Trials for Multiple Myeloma
Source: JAMA Netw Open. 2023 Nov 10;6(11):e2342195. doi: 10.1001/jamanetworkopen.2023.42195 (PMC10638643; doi:10.1001/jamanetworkopen.2023.42195)
Supplement: Supplement 2. — Data Sharing Statement [file jamanetwopen-e2342195-s002.pdf]

Data Sharing Statement

Najjar. Adverse Event Reporting in Randomized Clinical Trials for Multiple Myeloma. *JAMA Netw Open*. Published November 10, 2023. doi:10.1001/jamanetworkopen.2023.42195

Data
Data available: No

Additional Information
Explanation for why data not available: Available upon reasonable request to corresponding author
